# Chronic scrotal pain in young adults

**DOI:** 10.1186/s13104-017-2590-0

**Published:** 2017-07-04

**Authors:** Misgav Rottenstreich, Yuval Glick, Ofer Natan Gofrit

**Affiliations:** 1 Medical Corps, Israel Defense Forces (IDFMC), Tel Aviv, Israel; 2 Department of Urology, Hadassah Hebrew University Medical Center, Jerusalem, Israel

**Keywords:** Testicular disease, Pelvic pain, Primary care physicians, Military medicine

## Abstract

**Objective:**

Chronic scrotal pain (CSP) is a common and well recognized symptom of young males presenting to primary care units. Historically, CSP is defined as a testicular pain lasting for over 3 months. However, its etiology and outcome are poorly understood and its management is largely empirical. This study was conducted to examine the frequency, spectrum of pathology and outcome of CSP among young adults.

**Results:**

The medical records of 382,036 young males were reviewed for anamnestic information, physical findings, primary care physician decisions, and final outcome. CSP, defined as scrotal pain longer than 14 days, was recorded in 3084 patients (0.8%). The total number of primary physician’s visits due to this complaint was 16,222, with a mean of 5.3 visits per patient (range 1–37). Varicocele was the most common physical finding (54.1%). Other common findings were inguinal hernia (4.5%), genital infection (4.3%), hydrocele (4.2%) and referred pain (3.3%). 252 patients (8.2%) underwent surgical treatment but orchiectomy was not necessary in any patient. In 34.4% no specific etiology could be found. Neither malignant tumors nor testicular torsion were diagnosed in any patient.

The prevalence of the diagnoses was similar between the different time groups—15–29 days, 30–59 days and more than 60 days. Considering the similar etiologies CSP over a wide spectrum of time we suggest defining CSP as testicular pain lasting longer than 14 days.

## Introduction

Chronic scrotal pain (CSP) is a common and frustrating complaint that interferes with daily function of young men. It can lead to long evaluation process and its treatment is often ineffective [1–3].

CSP is historically defined as an intermittent or constant testicular pain, unilateral or bilateral, lasting for over 3 months that interferes significantly with the patients daily activities [4]. This CSP’s definition was stamped by Davis et al. in 1990, after conducting a retrospective study on 45 patients who approached the clinic with testicular pain lasted at least 3 months [4]. This definition was made according to common description of chronic pain as pain lasting usually 3–6 months [5]. However, chronic duration of pain is defined differently in different organs [6]. The prevalence of CSP and its spectrum of etiologies were never reported in the literature. Hence, this arbitrary definition might be inaccurate given the diagnoses’ medical course which causing CSP.

The management of CSP depends on the specific diagnosis or presumed etiology. Therefore, a thorough medical history, physical examination, laboratory tests (blood and urine investigations), imaging studies and specific tests like tumor markers and sexual transmitted disease screening, when indicated are crucial.

The literature is poor in epidemiological data on CSP, its etiologies, prevalence and outcome. Data from questionnaires among urologists in Switzerland revealed a crude incidence of about 350–450 cases of CSP per 100,000 men between 25 and 85 years [7]. Recent data from orchialgia clinic located at Mount Sinai Hospital in Toronto, Canada of 131 men presenting with CSP found that in 43.5% the cause was unknown; Vasectomy was found as the cause of the pain in 20.6%, trauma in 12.2% and infection in 11.5% [8].

The aim of this study is to provide a comprehensive epidemiologic description of CSP using a large database and to challenge the current definition of CSP.

## Main text

### Patients and methods

At age 18, most healthy Israeli males begin three years of mandatory military service. Men diagnosed with significant chronic medical or mental illness are not drafted.

Military recruitment can be delayed for a few years for high academic or religion studies. Soldiers who suffer from medical problem are treating by the general military clinic and being refer to a medical specialist as necessary. According to the Israeli military’s commands every medical visit is recorded in the medical corps database.

The computerized medical corps database of all referrals to general practitioners from: January 2004 to December 2014 and the medical records were searched using the key words: pain, testis, varicocele and orchialgia. All patients in current study were treated initially by general practitioners and were referred to further evaluation as needed. Patients with testicular pain lasting longer than 2 weeks were included in the study. Patients with prior genital or hernia surgery were excluded.

The patients were divided to three groups according to their symptoms duration: Group A—patients with symptom duration between 15 and 29 days, Group B—patients with symptom duration between 30 and 59 days, and Group C—patients with symptom duration above 60 days.

The study was approved by the Israel army’s Institutional Review Board.

### Diagnoses

Varicocele, hernia, hydrocele, skin lesion, torsion–detorsion and nephrolithiasis were diagnosed only after the approval of the physical examinations’ findings by ultrasonography. For statistical reasons, all genital tract infections were included together under one title of “genital infections”. Genital infections were diagnosed following positive urine or sperm culture or polymerase chain reaction (PCR) tests. The diagnoses of referred pain from the groins or adductor muscles were given after suitable findings in physical examination. The diagnoses of trauma to the scrotum were given upon patients’ report. Diagnosis of idiopathic scrotal pain was assigned when there were no clinical findings on physical examination, laboratory tests and ultrasonography.

### Statistical analyses

Statistical analysis was done using the Statistical Analysis System (SAS, Cary, North Carolina), with standard univariate analysis using unpaired t-tests for comparison of the means of groups. Various frequencies of diagnoses were compared with the Chi square or the Fisher exact tests. To determine the interdependence of univariate analysis, multiple logistic regression models of the variables that were significant on univariate analysis were used. Differences were considered statistically significant when the p value was less than 0.05.

## Results

### Patient characteristics

The medical records of 382,036 young males were reviewed, and 3084 patients (0.8%) with CSP were identified. 198 (6.4%) patients had symptom duration between 15 and 29 days (group A), 696 (22.6%) with symptom duration between 30 and 59 days (group B) and 2190 (71%) with symptom duration above 60 days (group C). Mean patient age was 19.24 years (range 18–27 years), mean weight was 67.6 kg and mean height was 174.8 cm.

### Primary physician visits and referrals

Data regarding primary physician visits and referrals for further evaluation is presented in Table 1. Total number of primary physician visits was 16,222 with mean visits of 5.3 per patient (range 1–37), mean number of visits among patients in group C (6.0) were significantly higher than in other groups (3.2 in group A and 3.6 in group B). Patients in group C were referred more often to ambulatory Urology clinics (at least once in 82% of the cases with a mean of 1.6 referrals per patient), ambulatory ultrasonography (82.4%), semen analysis (39.8%) and varicocele, hernia and hydrocele surgery (10.2%) compared to patients in groups A and B. A total of 252 patients (8.2%) underwent surgical treatment, 206 (6.7%) had varicocele, and/or hydrocele surgery and 46 (1.5%) hernia surgery. The rates of emergency department (ED) referrals were similar between groups A and C but were higher than group B. Hospitalization rates were similar between the groups.

**Table 1:** Referral of patients

	Group A—15–29 days			Group B—30–59 days			Group C—above 60 days			Total
Patients (n)	198	p value		696	p value		2190	p value		3084
Primary physician visits										
Total visits	633			2535			13,054			16,222
Mean	3.2	Gr. B	0.370	3.6	Gr. A	0.370	5.96	Gr. A	*0.000*	5.3
Range	1–11	Gr. C	*0.000*	1–13	Gr. C	*0.000*	1–37	Gr. B	*0.000*	1–37
Referrals										
Urologist specialist	57.1%	Gr. B	0.095	67.1%	Gr. A	0.095	82.0%	Gr. A	*0.000*	77.0%
		Gr. C	*0.000*		Gr. C	*0.000*		Gr. B	*0.000*	
Ultrasonography	70.2%	Gr. B	0.662	77.2%	Gr. A	0.662	82.4%	Gr. A	*0.000*	80.4%
		Gr. C	*0.000*		Gr. C	*0.000*		Gr. B	*0.000*	
Semen analysis	16.2%	Gr. B	0.178	24.0%	Gr. A	0.178	39.8%	Gr. A	*0.000*	34.7%
		Gr. C	*0.000*		Gr. C	*0.000*		Gr. B	*0.000*	
Emergency department	26.3%	Gr. B	0.954	22.8%	Gr. A	0.954	26.8%	Gr. A	0.896	25.9%
		Gr. C	0.896		Gr. C	*0.035*		Gr. B	*0.035*	
Hospitalization rate	1.5%	Gr. B	0.867	0.7%	Gr. A	0.867	1.0%	Gr. A	0.961	1.0%
		Gr. C	0.961		Gr. C	0.967		Gr. B	0.967	
Surgery	3.0%	Gr. B	1.000	3.3%	Gr. A	1.000	10.2%	Gr. A	*0.000*	8.2%
		Gr. C	*0.000*		Gr. C	*0.000*		Gr. B	*0.000*	

*Gr.* comparison with other group

### Diagnoses

Table 2 shows the final diagnoses. Varicocele (54.2%), idiopathic scrotal pain (34.4%), hernia (4.5%), genital infection (4.3%), hydrocele (4.2%) and referred pain from the groins or adductor muscles (3.3%) were the most common diagnoses. Varicocele was significantly more common in patients with longer symptom duration, 28.8% in group A, 41.1% in group B and 60.6% in group C (p < 0.0001). Reciprocally, the number of patients with idiopathic scrotal pain was significantly lower among patients with longer symptom duration (57.1% in group A, 45% in group B and 29% in group C, p = 0.012).

**Table 2:** Diagnosis

	Group A—15–29 days			Group B—30–59 days			Group C—above 60 days			Total
Patients (n)	198	p value		696	p value		2190	p value		3084
Varicocele	28.8%	Gr. B	*0.007*	41.1%	Gr. A	*0.007*	60.6%	Gr. A	*0.000*	54.1%
		Gr. C	*0.000*		Gr. C	*0.000*		Gr. B	*0.000*	
Idiopathic scrotal pain	57.1%	Gr. B	*0.012*	45.0%	Gr. A	*0.012*	29.0%	Gr. A	*0.000*	34.4%
		Gr. C	*0.000*		Gr. C	*0.000*		Gr. B	*0.000*	
Hernia	2.5%	Gr. B	0.322	4.6%	Gr. A	0.655	4.6%	Gr. A	0.573	4.5%
		Gr. C	0.573		Gr. C	1.000		Gr. B	1.000	
Genital infections	5.6%	Gr. B	1.000	5.8%	Gr. A	1.000	3.7%	Gr. A	0.789	4.3%
		Gr. C	0.789		Gr. C	0.207		Gr. B	0.207	
Hydrocele	5.6%	Gr. B	0.303	2.7%	Gr. A	0.303	4.6%	Gr. A	0.957	4.2%
		Gr. C	0.957		Gr. C	0.122		Gr. B	0.122	
Referred pain	3.5%	Gr. B	0.971	2.7%	Gr. A	0.971	3.4%	Gr. A	1.000	3.3%
		Gr. C	1.000		Gr. C	0.856		Gr. B	0.856	
Skin lesion	1.0%	Gr. B	1.000	1.0%	Gr. A	1.000	1.1%	Gr. A	0.999	1.1%
		Gr. C	0.999		Gr. C	0.991		Gr. B	0.991	
Torsion–detorsion	0.0%	Gr. B	0.992	0.1%	Gr. A	0.992	0.7%	Gr. A	0.972	0.5%
		Gr. C	0.972		Gr. C	0.999		Gr. B	0.999	
Trauma	0.5%	Gr. B	0.997	0.1%	Gr. A	0.997	0.2%	Gr. A	0.998	0.2%
		Gr. C	0.998		Gr. C	1.000		Gr. B	1.000	
Nephrolithiasis	0.5%	Gr. B	0.634	0.0%	Gr. A	0.634	0.1%	Gr. A	0.730	0.1%
		Gr. C	0.730		Gr. C	0.990		Gr. B	0.990	

*Gr.* comparison with other group

The prevalence of the other diagnoses was similar between the different groups.

Diagnosis of scrotal pain due to scrotal skin lesions, torsion–detorsion syndrome, scrotal trauma, scrotal tumors or nephrolithiasis were assigned in about 1% of patients or less. Malignancy and torsion of testis were assigned in none.

## Discussion

CSP is common and frustrating complaint of young adults. In this research, a large database was surveyed and the authors had full access to the long term outcome of the cases, so the risk of missing important diagnoses is low. To the best of our knowledge, this is the first large scale survey of CSP in young men in a primary care setting. It was found that CSP affects 0.8% of the young men. The mean primary physician’s number of visits was 5.3 with one patient visiting the clinic 37 times. Referrals to specialist in Urology were also common and the mean number of referrals was 1.4 per patient (range 1–11).

The etiologies of CSP in young men are reported in this study (Table 2). Varicocele was found in 54.6% of the patients and was more common among patients with longer duration of symptoms (up to 60.6% in group C). The prevalence of varicocele in normal young men estimated at 15–20% [9] and the prevalence of pain in individuals with varicocele is estimated between approximately 2 and 10% [10], which mean estimated prevalence of painful varicocele of 0.3–2% in normal young men population. We found that painful varicocele was found in 0.4% of normal young men population which is close to the expected rate.

According to Granitsiotis et al. nearly 25% of patients with chronic orchialgia have no obvious cause for the pain [1]. In our study, no specific etiology could be established in 1062 patients (34.4%). The percentage of patients with idiopathic scrotal pain dropped with longer symptom duration. This may suggests that in patients with idiopathic scrotal pain who complain for longer periods of time, further evaluations were done and in most cases, varicocele was found and diagnosed as the presumed cause of the symptoms.

The prevalence of the other diagnoses was quite similar between the different time groups from the shortest duration of pain (15–29 days—group A) to longest (more than 60 days—group C). This challenges the historic definition of CSP as pain lasting more than 90 days that is based on a study of 45 patients [4]. Therefore, we suggest defining CSP as an intermittent or constant testicular pain lasting for more 14 days that interferes significantly with the patient’s daily activities.

Diagnosis of scrotal pain due to scrotal skin lesions, torsion–detorsion syndrome, scrotal trauma, scrotal tumor or nephrolithiasis were assigned in about 1% of patients or less, probably because these etiologies are of acute manifestation or because of the painless nature of these pathologies. A total of 252 (8.2%) patients underwent surgical interventions including: hernia surgery in 46 patients (1.5%) and varicocele and/or hydrocele repair in 206 patients (6.7%). The rates of varicocele and/or hydrocele surgery were higher in group C, probably because of failure of conservative treatments.

The management of CSP depends on the cause or presumed etiology of the testicular pain. The key to successful assessment remains the history and physical examination and early use of ultrasound of testes and inguinal region is the most reliable imaging modality in the management of chronic testicular pain. Lau et al. observed that in patients with testicular pain longer than 14 days, sonography detected lesions in 28% of patients with no clinical findings on examination including varicocele, hydrocele, epididymal thickening and epididymal cyst [11]. Thus, we suggest early referral to ultrasonography of the testes and inguinal region for all patients with CSP.

Testicular torsion (TT) is a relatively rare urological emergency in which the diagnosis must be made accurately and rapidly to prevent loss of testicular function. TT is a medical situation of an acute presentation. However, TT may occur in patients with CSP. In current study, 25.9% of the patients visit at least once in the ED, in most cases because of physicians’ suspicious of TT. Yet TT and torsion of the appendix testes were not found in any of the patients and only 1.0% of the patients were hospitalized emergently for any reason.

## Conclusions

We report on the prevalence, etiologies and outcome of CSP in young men in a primary care clinic are reported. CSP affects almost 1% of the young males and can lead to repeated visits to the primary care clinic. Varicocele is found in half of the patients but significant pathology (tumor or torsion) are rare. Early use of ultrasonography is advised. It can rule out significant pathology, reassure the patient that the condition is benign and prevent revisits to the clinic. Considering the similar etiologies with minor variations of CSP over a wide spectrum of time (from pain longer than 2 weeks to pain longer than 60 days) we suggest defining CSP as intermittent or constant testicular pain lasting longer than 14 days that interferes significantly with the patient’s daily activities.

## Limitations

First, our database is obtained from medical registries of the Israeli army and it may be difficult to generalize the finding of this study to the general population. This issue may have affected our results; however, military service in Israel is a compulsory and includes not only combat healthy soldiers but also non-active ones which their life style is similar to a civilian life. Another concern is the fact that the study was done retrospectively with no control group, further prospective studies in civilian population using this study findings are needed. The absence of validated questioners regarding quality of life makes it impossible to evaluate the real impact of CSP on quality of life.

## Abbreviations

CSP: chronic scrotal pain
TT: testicular torsion
ED: emergency department
kg: kilograms
cm: centimeters
